# Next generation validation for next generation risk assessment

**DOI:** 10.3389/ftox.2026.1790669

**Published:** 2026-05-01

**Authors:** Karolina Kopańska, Thomas Hartung

**Affiliations:** 1 Bloomberg School of Public Health and Whiting School of Engineering, Center for Alternatives to Animal Testing (CAAT), Johns Hopkins University, Baltimore, MD, United States; 2 CAA Tevents, Solingen, Germany; 3 Doerenkamp-Zbinden Chair for Evidence-Based Toxicology, Baltimore, MD, United States; 4 CAAT-Europe, University of Konstanz, Konstanz, Germany

**Keywords:** artificial intelligence, e-validation, integrated testing strategies, mechanistic validation, new approach methodologies, next-generation risk assessment, toxicology, validation

## Abstract

Next generation risk assessment (NGRA) demands a fundamental transformation in how toxicological test methods are validated. Traditional validation approaches, designed for animal tests and mainly for simple *in vitro* methods, are increasingly inadequate for evaluating complex New Approach Methodologies (NAMs), artificial intelligence (AI)-based approaches, and integrated testing strategies (ITS). This paper presents a comprehensive framework for “*next-generation validation*” that leverages artificial intelligence and modern computational capabilities to create more efficient, thorough, and dynamic validation processes. The proposed framework emphasizes human relevance over simple concordance with animal data and emphasizes key innovations including e-validation, mechanistic validation, and post-validation companion AI agents. Because AI can inherit biases, obscure failure modes, and drift over time, the framework treats AI as both a tool for, and a subject of, validation, requiring transparent performance criteria, uncertainty quantification, and explicit governance for model updates and lifecycle monitoring. To make the framework actionable, we define a method as “*NGV-validated for a stated context of use*” when it meets pre-specified acceptance criteria across five domains, i.e., technical reliability, biological relevance, predictive performance, uncertainty quantification, and data integrity, supported by defined governance roles, version control, and lifecycle re-review triggers. e-validation employs sophisticated algorithms for reference chemical selection, study simulation, and continuous performance monitoring, while mechanistic validation evaluates whether methods accurately capture relevant biological pathways and mechanisms of toxicity. The paper addresses critical implementation challenges including data quality standardization, regulatory acceptance, and international harmonization, providing specific recommendations for various stakeholders. Looking forward, validation will increasingly embrace dynamic, adaptive approaches that evolve alongside scientific understanding and technological capabilities. The integration of artificial intelligence will enhance analysis of complex data, enable real-time monitoring of method performance, and support more sophisticated uncertainty quantification. Success in this transformation requires coordinated effort across regulatory agencies, industry partners, and academic institutions. In summary, this paper emphasizes a five-pillar framework integrating mechanistic, probabilistic, and AI-driven elements to reform toxicological validation. The proposed framework, exemplified here for tests for developmental neurotoxicants and virtual control groups, represents a crucial step toward more efficient and accurate chemical safety assessment while maintaining necessary standards for public health protection.

## 1 Introduction

Next-Generation Risk Assessment (NGRA) is an exposure-led, hypothesis-driven approach to evaluating chemical safety that integrates *in silico, in chemico*, and *in vitro* New Approach Methodologies (NAMs). NGRA aims to protect human health without relying on animal testing, focusing on real-world exposure scenarios, mechanistic understanding, and human relevance (Baltazar et al., 2020; Wood et al., 2024; Leso et al., 2025). A number of case studies for NGRA are available (Alexander-White et al., 2022; Ebmeyer et al., 2024; Gilmour et al., 2022; Luijten et al., 2020; Pallocca et al., 2022). A core principle is that if no bioactivity is observed at human-relevant exposure levels, adverse health effects can be excluded. This represents a shift away from extrapolating high-dose animal outcomes toward decision-making grounded in low, human-relevant exposures over realistic durations. Given the potentially large consequences of regulatory decisions, confidence in method selection and transparent justification for transitions from legacy to novel approaches are essential (van der Zalm et al., 2022).

The inevitable risk of bias (Hartung et al., 2025a) in toxicology requires careful assessment, i.e., validation. Validation of toxicological test methods (Hartung, 2007; Leist et al., 2012; Hartung, 2024) stands at a crossroads as the field moves towards NGRA (Hartung, 2025), where decisions increasingly rest on integrated evidence streams rather than single apical animal outcomes.

Traditional validation approaches, designed primarily for animal tests and mostly for simple *in vitro* methods, are increasingly inadequate for evaluating complex NAMs, including human-cell based microphysiological systems (MPS) (Marx et al., 2016; Marx et al., 2020; Marx et al., 2025; Nitsche et al., 2022; Hartung and Smirnova, 2025), artificial intelligence (AI)-based approaches (Hartung, 2023a; Hartung, 2023b; Kleinstreuer and Hartung, 2024), and integrated testing strategies (ITS) (Hartung et al., 2013a; Rovida et al., 2015; Caloni et al., 2022). Decade-long validation processes are no longer sustainable in an era that demands timely assessment of thousands of chemicals while maintaining public-health protection. Moreover, simple concordance with animal data is a weak anchor because many animal tests were never validated for human relevance, and traditional validation often freezes a method at a single point in time despite rapid technological evolution.

Importantly, AI is not a shortcut to certainty. AI-assisted validation can inherit or amplify biases in training data, obscure failure modes, and drift over time if models, code, or input data change. Next-Generation Validation (NGV) therefore treats AI as both a tool and a subject of validation, requiring transparent performance criteria, uncertainty quantification, and explicit governance for model updates and lifecycle monitoring.

The need for modernizing validation approaches has never been more pressing. The toxicology field is undergoing a fundamental transformation and this evolution demands validation procedures that can evaluate not just individual tests but complex methodological frameworks. Modern validation must be faster, more flexible, and better aligned with regulatory needs while maintaining scientific rigor. It must also address the growing importance of mechanistic understanding, biological relevance, and uncertainty quantification in risk assessment. NGRA demands validation procedures that can evaluate complex data integration, assess the biological relevance of predictions, and provide clear uncertainty quantification.

This article aims to outline a framework for NGV that aligns with and enables NGRA. We will explore how modern validation can evolve to meet current challenges while maintaining scientific rigor and regulatory acceptance. By examining novel approaches such as e-validation (Hartung et al., 2024b), mechanistic validation (Hartung et al., 2013b), and biomarker-based strategies (Hartung et al., 2024a), we propose a path forward that can accelerate the adoption of innovative methods while ensuring their reliability for safety assessment. The article will address key considerations including human relevance, technological integration, uncertainty quantification, and regulatory acceptance, providing practical recommendations for implementing next-generation validation approaches.

Through this examination, we seek to contribute to the ongoing transformation of toxicology and risk assessment, ensuring that validation evolves alongside the methods it evaluates. The ultimate goal is to enable more efficient, accurate, and human-relevant safety assessment while maintaining the high standards necessary for public health protection. Table 1 summarizes the core innovations of NGV in toxicology.

**TABLE 1:** Summary of core innovations of NGV in toxicology.

Innovation	Definition	Key features	Benefits
Mechanistic validation	Evaluation of methods based on their ability to reflect known biological mechanisms and pathways	• Anchored in adverse outcome pathways (AOPs) • Utilizes mechanistic biomarkers	• Enhances human relevance • Reduces reliance on animal concordance
e-validation	AI-enhanced, modular, and dynamic validation framework using simulations and real-time data	• Smart reference chemical selection • Simulation of study designs • Dashboard coordination	• Reduces validation time and cost • Improves study design and adaptability
AI companion agents	Post-validation artificial intelligence systems that monitor and update method performance	• Real-time performance tracking • Continuous learning • Alert and update capabilities	• Maintains method currency • Enables adaptive validation over time
Probabilistic assessment	Incorporation of uncertainty quantification and probabilistic reasoning into validation	• Confidence intervals and error analysis • Support for probabilistic risk assessment	• Increases transparency • Better decision support for regulators
Fit-for-purpose validation	Tailored validation requirements based on method application context	• Context-specific performance criteria • Scalable rigor based on intended use	• Optimizes resource use • Enables broader implementation of NAMs

## 2 Addressing bias through next-generation validation

Toxicological studies are the cornerstone of chemical safety, yet they remain susceptible to systematic errors that can undermine public health protection. It is crucial to distinguish between “*risk of bias*” – defined as systematic errors in the design or conduct of a specific study, - and “*method validation*,” which evaluates the fundamental reliability and relevance of the test method itself.

Validation serves as the primary firewall against poor science. By enforcing standardization - recently enhanced by Good Cell and Tissue Culture Practice (GCCP 2.0) (Pamies et al., 2020; 2022) and Good *In Vitro* Reporting Standards (GIVReSt) (Hartung et al., 2019; Mohapatra et al., 2025; Mohapatra and Hartung, 2026), validation also minimizes variability. Furthermore, by anchoring methods to validated biomarkers of effect, it links *in vitro* results to relevant biological outcomes, reducing subjectivity. However, the validation process itself is not immune to bias. Traditional validation frameworks face inherent vulnerabilities that NGV must address through AI and computational integration.

### 2.1 Selection bias: the reference chemical trap

Selection bias in validation often manifests through the non-representative selection of reference compounds, with an overemphasis on well-characterized chemicals that are “easy” to test. This results in limited chemical space coverage and potentially optimistic performance estimates that fail when applied to novel chemistries.

- Next-Generation Mitigation: AI algorithms can now systematically analyze structural features and physicochemical properties to identify gaps in reference sets (Hoffmann et al., 2008). e-validation frameworks employ these algorithms to curate “*smart*” reference sets that ensure broad, unbiased coverage of the applicability domain.

### 2.2 Performance bias: the reproducibility crisis

Performance bias emerges from variations in experimental conditions, operator expertise, and equipment calibration across laboratories. In complex NAMs, such as high-content imaging or organ-on-chip systems, slight deviations in protocols can introduce systematic disparities.

- Next-Generation Mitigation: e-validation moves beyond static protocols to dynamic simulation. By simulating study designs and parameter combinations before physical testing, researchers can identify robust experimental validation conditions. Furthermore, AI-driven monitoring can detect subtle drift patterns, e.g., “*plate effects,*” in real-time that human operators might miss, ensuring consistency across diverse laboratory settings.

### 2.3 Detection and reporting bias: the “black box” problem

Subjective interpretation of endpoints (Detection Bias) and the selective publication of favorable results (Reporting Bias) pose significant threats to validation integrity. The omission of failed validation attempts or optimization steps obscures critical limitations, leading to an overestimation of a method’s readiness.

- Next-Generation Mitigation: The integration of automated documentation systems and machine-readable formats facilitates objective quality checks. AI systems can verify adherence to reporting templates (such as GIVReSt) and flag inconsistencies, ensuring that method limitations are as transparent as their successes.

By acknowledging these biases and deploying AI-facilitated mitigation strategies, Next-Generation Validation does not just evaluate methods; it actively improves them. This shift from a static “*pass/fail*” exercise to a dynamic, bias-aware framework is essential for the regulatory acceptance of complex NAMs.

## 3 The evolution of validation

The validation of toxicological methods has undergone significant evolution since its formal inception in the 1980s. Initially developed through workshops at the Center for Alternatives to Animal Testing (CAAT) and later refined through international collaboration, traditional validation approaches established foundational principles for evaluating new test methods (Hartung, 2024). These early frameworks focused primarily on reproducibility and relevance assessed a prediction of the results of animal studies, employing rigid, step-wise processes that often spanned a decade or more from method development to regulatory acceptance (see Ferrario et al., 2014, for definitions). While these approaches successfully validated several alternative methods, their limitations have become increasingly apparent in the modern era of toxicology.

Traditional validation faces several fundamental constraints that hinder innovation in toxicology. The process typically requires extensive ring trials involving multiple laboratories, demanding substantial resources and time. The reliance on animal data as the “*gold standard*” for comparison perpetuates the use of methods that themselves have never been formally validated for human relevance. Furthermore, these approaches struggle to accommodate complex endpoints, integrated testing strategies, and rapidly evolving technologies. The static nature of traditional validation, which effectively freezes method protocols at a point in time, poorly serves the dynamic nature of modern toxicology.

A key conceptual advance in the evolution of validation was the modular approach introduced by the European Centre for the Validation of Alternative Methods (ECVAM) in 2004, which proposed decomposing validation into independently assessable components such as test definition, transferability, predictive capacity, and performance standards (Hartung et al., 2004). This framework allowed for greater flexibility by enabling a mix of prospective and retrospective validation and laid the groundwork for adaptive mechanisms such as “*catch-up validation*.” Importantly, it emphasized that validation is not a monolithic exercise but a structured evaluation of reliability and relevance tailored to the method’s context of use. The modular approach anticipated many challenges now addressed by NGV, such as the need to accommodate new technologies (e.g., Quantitative Structure-Activity Relationships (QSARs), ∼omics, AI) and to reassess validated methods as scientific understanding evolves. Our proposed e-validation framework builds directly on this foundation, using AI and continuous monitoring to operationalize modular reassessment in real time and at scale. Griesinger et al. (2016) formalized a comprehensive typology of validation pathways, including prospective, retrospective, performance-standard-based, and evidence-based validation. Their work advanced the modular validation paradigm by integrating statistical rigor, mechanistic justification, and practical constraints such as resource availability and high-throughput platform deployment. Importantly, the authors emphasized that validation is not a monolithic or one-time exercise but a dynamic process that must evolve with scientific progress, regulatory context, and technological capabilities. Their treatment of relevance as a multidimensional construct, incorporating biological plausibility, predictive capacity, and applicability domain has shaped the way NGV integrates into complex testing frameworks such as Integrated Approaches to Testing and Assessment (IATA) (Tollefsen et al., 2014). This foundational work aligns with our proposal for adaptive, AI-enabled validation systems and supports the shift toward flexible, context-specific assurance of method performance.

A major contribution to the conceptual evolution of validation was the ECVAM Workshop report by Balls et al. (2006), which established a structured framework for validation based on Weight-of-Evidence (WoE) approaches. This method enables evaluation of test methods or strategies through structured, independent assessment of existing data - without requiring new ring-trial experiments. The authors outline clear criteria for test readiness, data inclusion, and procedural transparency, aligning WoE validation with principles from evidence-based medicine and meta-analysis. Importantly, the framework anticipates the validation needs of integrated approaches and testing strategies, recognizing that many future NAMs will not function as stand-alone guideline replacements. This approach provides a solid foundation for our proposal of dynamic, AI-assisted e-validation systems that combine prospective and retrospective elements within a continuously monitored validation lifecycle.

Our 2010 conceptual paper (Hartung, 2010) reframed validation for the 21^st^ century by introducing Evidence-Based Toxicology (EBT), drawing methodological tools and governance principles from Evidence-Based Medicine (Hartung, 2024; Hartung and Tsaioun, 2024). EBT promotes systematic reviews, probabilistic reasoning, and mechanistic anchoring *in lieu* of rote comparison to outdated animal models. Crucially, the article advocates for “*mechanistic validation*” (Hartung et al., 2013b) via pathway-level confirmation and probabilistic performance metrics rather than binary concordance. It also outlines the validation of Integrated Testing Strategies and calls for a global Human Toxome mapping effort (Hartung and McBride, 2011) to support what is now called NGRA. These principles resonate strongly with our proposal for e-validation (Hartung et al., 2024b), particularly in supporting dynamic, AI-assisted systems that quantify uncertainty, update predictions iteratively, and prioritize scientific relevance over legacy consensus.

Judson et al. (2013) make a compelling case for streamlining validation requirements for high-throughput screening (HTS) assays used in chemical prioritization. They propose a shift from traditional multi-laboratory ring trials to performance assessments based on large-scale reference compound testing and automated statistical reproducibility analysis. Notably, they redefine validation criteria in the context of prioritization rather than definitive hazard determination, emphasizing assay fitness-for-purpose, relevance to molecular key events, and intra-laboratory reproducibility. Their rolling validation model, illustrated as an iterative loop integrating development, use, and refinement, closely parallels our proposal for adaptive e-validation and AI-supported monitoring. This work supports the argument that rigid, one-size-fits-all validation pipelines are insufficient for modern, scalable approaches to toxicology.

Current challenges in validating NAMs have highlighted the need for further evolution in validation strategies, as they often employ complex biological systems, high-throughput technologies, and computational approaches that traditional validation frameworks struggle to evaluate. The integration of AI, MPS, and ∼omics technologies presents novel validation challenges. These methods may provide mechanistic insights and human-relevant data that cannot be directly compared to traditional animal studies. Additionally, the rapid pace of technological advancement means that validation processes must become more agile to prevent approved methods from becoming outdated before they reach widespread use.

The field now stands at a point requiring a fundamental paradigm shift in validation (Sewell et al., 2024). This transformation must move beyond simple correlation with animal data to embrace a more sophisticated understanding of biological relevance and human applicability. Modern validation needs to accommodate the dynamic nature of new technologies while maintaining scientific rigor. It must balance the need for thorough evaluation with the urgency of implementing improved methods for chemical safety assessment. The paradigm shift should incorporate mechanistic understanding, enable the evaluation of integrated approaches, and provide frameworks for continuous improvement and updates to validated methods.

This evolution demands new approaches that can evaluate the complex, multi-faceted nature of modern toxicology methods. Validation must expand to assess not just individual tests but integrated testing strategies and decision frameworks. It must embrace uncertainty quantification and probabilistic approaches to better reflect the reality of biological systems. The new paradigm should also consider the role of AI both as a subject of validation (Hartung and Kleinstreuer, 2025) and as a tool to enhance the validation process itself (Hartung et al., 2024b). The transformation of validation represents a critical challenge for advancing toxicology and risk assessment. Success requires balancing innovation with rigor, speed with thoroughness, and flexibility with standardization. The next-generation of validation must build on historical principles while embracing new technologies and approaches to meet the demands of modern toxicology. This evolution is essential for enabling the implementation of more efficient, human-relevant approaches to chemical safety assessment while maintaining public health protection.

## 4 Key components of next-generation validation

**FIGURE 1:**
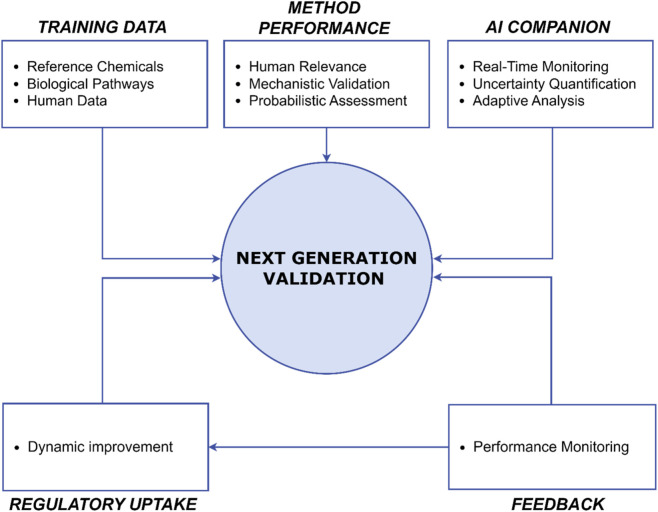
Elements of the next-generation validation framework.

Next-Generation Validation represents a fundamental reimagining of how we evaluate new toxicological methods, built on several interconnected components that together enable more efficient, relevant, and scientifically robust validation (Figure 1). At its core, this new approach prioritizes human relevance over simple concordance with animal data, recognizing that the ultimate goal of toxicological assessment is to protect human health. This shift requires moving beyond traditional one-to-one comparisons with animal studies to embrace a more sophisticated understanding of human biology and toxicological mechanisms.

The emphasis on human relevance manifests through deeper integration of human-derived data and mechanistic understanding. Rather than treating the validation process as a simple comparison of results, next-generation validation examines whether methods accurately capture relevant biological pathways and mechanisms of toxicity. This mechanistic validation approach evaluates whether test methods reflect known biological processes and AOPs (Leist et al., 2017). By anchoring validation in mechanistic understanding, we can better assess whether methods provide meaningful insights into human toxicological responses.

Biomarker-based approaches represent another crucial component of NGV (Hartung et al., 2024a). By incorporating validated biomarkers of toxicity and effect, validation can better link test method results to relevant biological outcomes (Blaauboer et al., 2012). These biomarkers serve as bridges between *in vitro* observations and *in vivo* effects, providing mechanistic context and enhancing the biological relevance of validation assessments. The integration of biomarkers also enables better translation between different test systems and ultimately to human outcomes.

Artificial intelligence and computational methods play dual roles in NGV. First, they serve as powerful tools for enhancing the validation process itself, enabling more sophisticated analysis of validation data and simulation of potential outcomes. Second, as AI-based methods increasingly contribute to toxicological assessment, validation approaches must evolve to effectively evaluate these computational approaches (Hartung and Kleinstreuer, 2025). This includes developing frameworks for assessing model transparency, reliability, and domain of applicability.

Performance-based evaluation (Holzer et al., 2023) represents a shift from rigid protocol-based validation to a more flexible approach focused on achieving defined performance standards. This component recognizes that different methods may achieve the same goal through various means. Rather than prescribing exact procedures, validation focuses on establishing clear performance criteria that methods must meet for their intended purpose. This approach enables innovation while maintaining scientific rigor.

The concept of “*fit-for-purpose validation*” acknowledges that different applications may require different levels of validation. A screening tool may not need the same degree of validation as a method used for final regulatory decisions. This approach allows validation efforts to be tailored to the specific context of use, enabling more efficient use of resources while ensuring appropriate rigor for critical applications. Fit-for-purpose validation also recognizes that methods may be valid for some applications but not others, allowing for more nuanced implementation of new approaches.

Uncertainty quantification and probabilistic approaches represent essential components of NGV. Moving beyond simple binary assessments of validity, these approaches provide a more nuanced understanding of method reliability and applicability. Probabilistic approaches allow for better characterization of uncertainty in test results and clearer communication of confidence levels in predictions (Paparella et al., 2013). This component aligns with modern risk assessment approaches that increasingly embrace probabilistic methods.

Integration of these components requires sophisticated frameworks for data analysis and decision-making. Modern validation must be able to synthesize multiple lines of evidence, from mechanistic data to performance metrics, into coherent assessments of method validity. This integration often employs weight-of-evidence approaches that can systematically consider diverse types of information while maintaining transparency in decision-making.

The implementation of these components demands new tools and approaches for validation management. This includes systems for tracking method performance over time, frameworks for updating validations as new data become available, and platforms for sharing validation information across stakeholders. Modern data management systems and collaborative platforms play crucial roles in enabling these capabilities.

Success in implementing these components requires careful attention to regulatory requirements and acceptance criteria, what we called earlier the “post-validation” process (Bottini et al., 2008). While NGV embraces new approaches and technologies, it must still provide the reliability and confidence needed for regulatory decision-making. This balance between innovation and regulatory acceptance represents an ongoing challenge in the evolution of validation approaches.

The key components of next-generation validation work together to create a more sophisticated, flexible, and scientifically robust approach to method evaluation. This system can better accommodate modern test methods while maintaining the rigor necessary for public health protection. As toxicology continues to advance, these components will likely evolve further, requiring ongoing adaptation of validation approaches to meet new challenges and opportunities.

### 4.1 Operationalizing NGV: defining “validated” for a context of use

To make NGV actionable, the validation question must be framed as: “Is this method sufficiently reliable and relevant for a specified context of use?” Accordingly, we define a method (or integrated workflow) as NGV-validated for a stated context of use when it meets pre-specified acceptance criteria across five domains: (1) technical reliability, (2) biological relevance/mechanistic anchoring, (3) predictive performance, (4) uncertainty quantification and applicability domain, and (5) data integrity and traceability. Crucially, these criteria must be set prospectively (ideally with regulators and end-users) and reported with uncertainty, rather than inferred *post hoc* from a single summary statistic.

Illustrative examples of quantitative and qualitative acceptance criteria (to be adapted fit-for-purpose) include:

- Reliability (within- and between-laboratory): pre-defined reproducibility targets for primary endpoints (e.g., ICC ≥0.75 and CV ≤ 20% across donors/labs), plus pre-registered control charts to detect drift.
- Predictive performance: for classification endpoints, external validation with sensitivity and specificity each ≥0.80 (or other agreed operating point), with AUROC/AUPRC and confidence intervals; for regression endpoints, pre-defined error bounds (e.g., RMSE not exceeding a specified fraction of biological variability).
- Calibration and uncertainty quantification: reported prediction intervals and calibration metrics (e.g., nominal 90% interval coverage within 85%–95%; calibration slope 0.8–1.2; Brier score/expected calibration error), enabling probabilistic decision-making rather than binary calls.
- Applicability domain: explicit definition (chemistry space and biology space); documented handling of out-of-domain inputs; evidence that the intended-use chemicals are predominantly in-domain (e.g., ≥90%).
- Data integrity and traceability: standardized metadata (e.g., ≥95% completeness for required fields), immutable dataset identifiers/checksums, and an audit trail linking raw data, preprocessing, model/version, and outputs.
- Lifecycle triggers: pre-defined re-review/revalidation triggers (e.g., performance drop >5% absolute on a sentinel set, detected covariate shift beyond threshold, code/library changes, or a new intended use).

The numerical thresholds above are examples meant to illustrate the type of decision-ready criteria NGV should require; actual acceptance criteria must be calibrated to the decision consequence (screening vs. regulatory restriction), baseline variability of the endpoint, and the available reference information.

### 4.2 Governance and documentation for lifecycle validation

Because many NAMs and AI models evolve (new training data, software updates, reagent changes, improved image pipelines), NGV must be supported by explicit governance. At minimum, this includes clearly assigned roles (method developer, independent validation/qualification body, data steward, and regulatory liaison), version control for code and data, and transparent procedures for change management. Recent regulatory thinking on AI in medicines emphasizes a risk-based approach, traceability, data governance, human oversight, and post-deployment monitoring (European Medicines Agency, 2024); these expectations map directly onto NGV for toxicological decision-making (Box 1).

We propose that an NGV dossier (submitted for regulatory review or public qualification) should, at a minimum, include:

- Context of use and decision consequence (what decision the method supports, and how uncertainty is handled).
- Method definition: SOPs and critical parameters (for assays), or architecture, features, and training procedure (for AI models), including a fixed version identifier.
- Data package: provenance, inclusion/exclusion rules, metadata completeness, and bias assessment of reference datasets; for AI, a “model card” plus dataset descriptors (analogous to QMRF/QPRF).
- Performance evidence: pre-registered metrics and acceptance thresholds, external validation results with uncertainty, and evidence for applicability domain boundaries.
- Mechanistic anchoring: AOP/MOA rationale and biomarker evidence showing biological plausibility for intended endpoints.
- Uncertainty quantification: calibration, prediction intervals, and sensitivity analyses (e.g., to donors, laboratories, or preprocessing choices).
- Change-control and monitoring plan: permitted updates, revalidation triggers, sentinel test sets/control charts, and reporting cadence (aligned with predetermined change control plans where applicable).

BOX 1 **Illustration of NGV evidence generated for regulatory confidence.**

Example evidence artifacts that an NGV-compliant workflow would generate include:

- a machine-readable assay/model description;
- a reference set rationale with chemical-space coverage plots;
- pre-registered analysis scripts and containers to ensure computational reproducibility;
- an external validation report with performance and calibration metrics plus uncertainty bounds;
- a mechanistic plausibility report (AOP/MOA mapping, biomarker concordance);
- an audit trail and dataset checksums;
- post-deployment monitoring dashboard with drift alerts and pre-defined revalidation triggers.

## 5 e-validation: a framework for AI-facilitated NAMs

e-validation represents a transformative approach to method validation, leveraging artificial intelligence and modern computational capabilities to create a more efficient, comprehensive, and dynamic validation process. As we recently outlined (Hartung and Kleinstreuer, 2025), this framework addresses the limitations of traditional validation while tackling the unique challenges posed by AI-based NAMs, such as data dependence and “black box” opacity.

While the vision of e-validation is future-facing, key elements are feasible today and can be implemented incrementally: algorithm-assisted reference selection, simulation-based power calculations, automated compliance checks (e.g., reporting standards), containerized deployments, and statistical drift monitoring. More autonomous components (e.g., “*companion agents*” that recommend updates) should be introduced under explicit human oversight and predefined change-control rules, so that adaptivity improves performance without eroding traceability or regulatory confidence.

The e-validation framework operates through several interconnected pillars:

### 5.1 Smart reference selection and data quality

Foundational to e-validation is the transition from expert-based to AI-driven reference chemical selection. Algorithms analyze chemical space, biological activity profiles, and toxicological data to identify the most representative test substances. This mitigates selection bias by ensuring coverage across diverse chemical classes while minimizing redundancy. For AI-facilitated NAMs, this process is inextricably linked to data quality. Since AI models are inherently dependent on training data, e-validation emphasizes the use of curated, standardized datasets that represent accurate and complete biological domains.

### 5.2 Simulation and *in silico* trials

e-validation utilizes advanced computational modeling to simulate validation study outcomes before physical implementation. By modeling various study designs and parameter combinations, researchers can identify potential pitfalls and optimize protocols, significantly reducing the resource burden of traditional ring trials.

### 5.3 Mechanistic AI and explainability

A critical barrier to regulatory acceptance of AI-based methods is their black box nature. e-validation addresses this by integrating Explainable AI (xAI) with mechanistic validation. Rather than relying solely on correlation, AI tools analyze vast biological pathway information to verify that the method captures relevant AOPs. This mechanistic anchoring provides the scientific confidence necessary for regulatory decision-making, ensuring that predictions are biologically plausible.

### 5.4 Reproducibility and dashboard coordination

Unlike traditional computational methods, AI-based NAMs are sensitive to software versions, computational architecture, and stochastic algorithms. e-validation manages these variables through centralized dashboard coordination. This intelligent interface acts as an operational hub, integrating automated quality checks, verifying consistency across computational environments, and managing data traceability via secure platforms.

### 5.5 Post-validation companion AI agents

Perhaps the most innovative aspect of e-validation is the introduction of post-validation companion AI agents. Traditional validation is a static “snapshot” in time; however, AI models evolve. These companion agents provide continuous, real-time monitoring of method performance after initial validation. They track predictive accuracy, alert users to domain shifts, and incorporate new data to suggest model updates. This dynamic approach, outlined in contrast to traditional methods in Table 2, ensures that validated methods remain current and reliable, transforming validation from a one-time event into a continuous lifecycle. Table 2 summarizes the fundamental shift from traditional QSAR validation principles to the requirements for AI-based models:

- Transparency: Shifts from explicit mathematical relationships to xAI tools.
- Reproducibility: Moves from fully defined algorithms to managing sensitivity in training data and software dependencies.
- Adaptability: Transitions from static validation to continuous performance tracking via AI agents.

**TABLE 2:** Comparison of the Established Validation Principles and the Needs for Validation of Emerging AI- based NAMs.

Validation aspect	Traditional QSAR (OECD principles)	AI-based model validation
Transparency	• Explicit mathematical relationship • Mechanistic interpretation often expected	• Often lacks interpretability (“black box”) • xAI tools needed
Reproducibility	• Fully defined algorithm and descriptors • High reproducibility across platforms	• Sensitive to training data, software libraries, and random seeds • Reproducibility varies
Adaptability over time	• Typically static once validated • No built-in learning or updating	• Inherently adaptive; can retrain on new data • Requires versioning and monitoring
Domain of applicability	• Defined via descriptor space or applicability domain analysis	• Often unclear or dynamically changing; needs probabilistic boundaries or confidence metrics
Training data standards	• Curated datasets with defined endpoints • Emphasis on experimental consistency	• Relies on large-scale heterogeneous data • Data curation and bias detection are critical
Validation metrics	• R^2^, root mean squared error (RMSE), external validation set, applicability domain	• Also includes precision, recall, area under the curve (AUC), and calibration curves • Uncertainty quantification essential
Regulatory acceptance	• Well-established OECD principles (2004, 2014) • Accepted in REACH, EPA, etc.	• Emerging; requires new guidance • Often accepted only as supporting evidence
Documentation	• Defined QSAR model reporting format (QMRF) • Clear descriptor and algorithm documentation	• Needs standardized model cards or documentation • Explainability tools are under development
Performance monitoring	• Typically no post-validation monitoring	• Enables continuous performance tracking (e.g., via AI companion agents)

**FIGURE 2:**
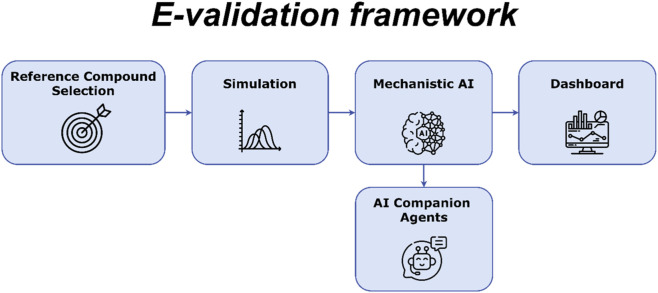
The e-Validation framework.

By integrating these components, E-validation (Figure 2) provides the sophisticated infrastructure needed to evaluate modern, complex NAMs while maintaining the scientific rigor required for public health protection.

## 6 Integration with next-generation risk assessment

NGV must seamlessly integrate with NGRA approaches to enable more efficient and accurate safety evaluations. This integration requires validation to support structured decision-making frameworks, where multiple evidence streams are combined and uncertainties are carried forward. Accordingly, validation approaches must evolve to support NGRA workflows such as the Alternative Safety Profiling Algorithm (ASPA) (Leist et al., 2026), which integrates NAM-derived evidence across exposure, kinetics, and hazard pillars. Rather than validating individual methods in isolation, NGV evaluates how methods perform as parts of an integrated workflow: how uncertainties combine, how conflicting evidence is resolved, and whether the overall decision logic remains robust under realistic variability. This shift from single-test validation to workflow validation is central to NGRA implementation.

The support of WoE approaches (Hoffmann et al., 2022) represents another crucial aspect of integration with NGRA. Modern validation frameworks must provide clear guidance on how different types of evidence can be weighed and combined. This includes developing standardized approaches for assessing data quality, relevance, and consistency across different methods and studies. Validation procedures need to evaluate not just the performance of individual methods but their contribution to overall weight of evidence assessments. This requires sophisticated frameworks for data integration and evidence synthesis that can handle diverse types of information while maintaining transparency in decision-making (Ouedraogo et al., 2025).

Enabling probabilistic risk assessment (Maertens et al., 2022; Maertens et al., 2024a; Maertens et al., 2025) represents a fundamental shift in validation approaches. Traditional validation often focused on binary outcomes, but NGRA demands more nuanced understanding of uncertainty and variability. Next generation validation must incorporate probabilistic methods that can characterize uncertainty in test results and support quantitative risk assessment. This includes developing frameworks for assessing and combining uncertainties across different test methods, understanding the impact of biological variability, and providing clear communication of confidence levels in predictions. Given the complexity of these tasks, external tools with integrated mathematical frameworks can support toxicologists in uncertainty quantification, result integration, and decision-making, yielding probabilistic outputs in an automated manner, as demonstrated by Kopańska et al. (2025). Such tools, however, would themselves require appropriate validation.

Real-time monitoring and updates become essential features of modern validation frameworks. As new data become available and scientific understanding advances, validation status must be continuously reassessed. This requires sophisticated systems for tracking method performance, identifying emerging issues, and updating validation assessments accordingly. AI can play a crucial role in this process, automatically analyzing new information and assessing its impact on method validity. This dynamic approach ensures that validated methods remain current and reliable over time.

Integration with exposure assessment represents another critical dimension of alignment with NGRA (Tkalec et al., 2024). Validation must consider how methods perform across relevant exposure scenarios and concentration ranges. This includes evaluating method sensitivity at environmentally relevant concentrations, understanding the relationship between *in vitro* concentrations and real-world exposures (Han et al., 2024), and assessing method performance for different exposure routes and patterns. The integration of exposure considerations into validation helps ensure that methods provide meaningful information for risk assessment.

Machine learning algorithms can help identify patterns and relationships in complex datasets (Ajisafe et al., 2025; Seal et al., 2025). Metamodels, which integrate multiple machine learning models trained on different datasets, are commonly used to analyze the combined effects of various mechanistic patterns for a specific chemical (Rodríguez-Belenguer et al., 2023). Based on the predicted results, artificial intelligence supports decision-making processes (Bhuller et al., 2025b). These tools can optimize testing strategies, predict method performance under different conditions, and support the interpretation of results within a risk assessment context.

Data management systems play a crucial role in enabling integration between validation and risk assessment. Modern platforms must support the seamless flow of information between different stages of assessment, ensuring that validation data and conclusions are readily available to risk assessors, as exemplified by Leist et al. (2026). This includes maintaining clear documentation of validation status (which includes versioning of methods), uncertainties, and limitations that can inform risk assessment decisions.

The regulatory context remains paramount in considering integration between validation and NGRA. Validation frameworks must evolve to support regulatory decision-making while accommodating new approaches to risk assessment. This includes developing clear criteria for method acceptance, establishing procedures for updating regulatory guidance, and ensuring transparency in decision-making processes (Hilton et al., 2023).

Training and education represent essential components of successful integration (McCarthy et al., 2025; Westmoreland et al., 2022). Stakeholders need to understand how validated methods fit within NGRA frameworks and how to interpret and use validation information appropriately. This requires development of comprehensive training programs and support materials that address both technical and practical aspects of method implementation.

International harmonization becomes increasingly important as validation integrates with NGRA. Different regulatory jurisdictions may have varying requirements and approaches to risk assessment, necessitating flexible validation frameworks that can accommodate different needs while maintaining scientific rigor. Collaboration between international organizations and regulatory bodies helps ensure consistent approaches to validation and risk assessment (Bottini et al., 2007).

Through careful attention to these various aspects of integration, NGV can effectively support the implementation of NGRA while maintaining the rigor necessary for regulatory acceptance. This evolution enables more efficient and accurate safety assessment while providing the flexibility needed to accommodate advancing scientific understanding and technological capabilities.

## 7 Implementation challenges and solutions

Despite the promise of NGV frameworks, realizing their full potential requires overcoming several interrelated scientific, regulatory, technical, and cultural challenges (Table 3). These must be addressed systematically to ensure successful implementation while upholding the principles of scientific rigor, transparency, and public health protection.

**TABLE 3:** Key implementation challenges and mitigation strategies.

Challenge	Description	Solutions
Data quality and standards	Toxicological data are diverse, complex, and often lack standardized formats, impeding integration and reuse	Adopt FAIR principles; establish ontologies, automated quality control, and shared data standards tailored for toxicology
Regulatory acceptance	Regulatory bodies are cautious toward unfamiliar, complex methodologies	Engage regulators early; provide transparent documentation and performance data; align with regulatory needs while maintaining scientific rigor
Resources and cost	High infrastructure and expertise demands may limit uptake, especially in smaller organizations	Leverage cloud-based platforms, open-source tools, and public-private partnerships; create validation centers of excellence to support shared resources and expertise
Training and expertise	Implementing new validation approaches requires cross-disciplinary knowledge and ongoing training	Develop interactive training platforms and competency assessments; use AI-assisted personalized learning tools; update content regularly to reflect new methods
International harmonization	Diverse national requirements can result in duplication and inefficiencies	Promote collaboration through international working groups; develop shared validation principles and mutual recognition frameworks
Technical infrastructure	Advanced validation requires scalable, secure, and interoperable IT systems	Build modular, cloud-ready platforms; enforce interoperability standards; ensure cybersecurity and data traceability
Change management	Transitioning from legacy systems and cultures to dynamic validation models can face internal resistance	Implement structured change management strategies, maintain continuity of operations, and foster a culture of innovation and learning

### 7.1 Challenges for implementing NGV

A key challenge is the limited explainability of advanced AI systems. Many state-of-the-art machine learning algorithms, particularly deep learning models, function as black boxes with opaque internal logic. This lack of transparency complicates regulatory review and stakeholder trust, especially in contexts where mechanistic understanding is essential. Progress in xAI offers potential solutions, but these are still maturing and not universally applicable.

Another significant limitation is the variable access to high-quality, curated training data, which remains fragmented across geographies and institutions. Legal constraints on data sharing, inconsistent formatting, and limited metadata often hinder the development of robust, generalizable models. Data sparsity is especially problematic for underrepresented endpoints, low-dose effects, and susceptible populations. Investment in federated data platforms and harmonized ontologies is urgently needed.

Moreover, regulatory harmonization has not kept pace with scientific innovation. While some agencies are pioneering NAM integration and adaptive guidance frameworks, others remain entrenched in legacy requirements centered around animal models. This heterogeneity risks undermining international coherence in chemical safety assessment and imposes additional burdens on global industry actors. A coordinated push for mutual recognition and convergence of validation criteria is critical.

To enable the transition from traditional to NGV, stakeholders must invest in foundational infrastructure, inclusive capacity building, and collaborative governance. Regulatory authorities should be involved early and actively in the design and evaluation of novel approaches to ensure relevance and acceptance. Simultaneously, incentives must be aligned to reward transparency, reproducibility, and continuous performance monitoring.

A phased implementation strategy, accompanied by regular evaluation and refinement, will help mitigate risks while capitalizing on technological advancements. Success depends not only on scientific and technical excellence, but also on fostering a shared vision across sectors and jurisdictions that places human relevance, efficiency, and ethical responsibility at the heart of toxicological innovation.

### 7.2 Regulating adaptive systems: from static guidelines to lifecycle management

A critical implementation challenge for NGV is the fundamental mismatch between dynamic AI-based methods and the current regulatory framework. Traditional OECD Test Guidelines (TGs) are legally and operationally “*static,*” meaning that once they are adopted, the protocol is frozen. Any modification typically triggers a multi-year re-validation and peer-review process. This rigidity is incompatible with AI-facilitated NAMs that are designed to learn, evolve, and improve over time (e.g., via “*continuous learning*” or companion AI agents). If a validated AI model updates its algorithm to incorporate new reference data, it technically becomes a “new” method, potentially voiding its regulatory acceptance. To overcome this, regulatory toxicology must move from certifying a fixed method to certifying a method lifecycle.

The solution: Predetermined Change Control Plans (PCCPs). Regulatory bodies can look to lifecycle-based oversight models from other regulated domains (e.g., AI/ML-enabled medical devices) as a practical blueprint. Under a PCCP, a developer submits not only the model/assay but also a prospective “change protocol” describing permitted updates, how updates will be verified, and when escalation to regulators is required. This lifecycle orientation is consistent with recent European regulatory thinking on AI (European Medicines Agency, 2024) and can be adapted to toxicology.

What modifications are allowed (examples): retraining on new reference data from pre-defined sources; updating preprocessing pipelines within validated bounds; or replacing software dependencies within a specified version range, while keeping the intended context of use unchanged.

How the method will be re-verified automatically (examples): re-running a locked “*sentinel*” test set and confirming that key metrics remain within bounds (e.g., AUROC and calibration error within pre-specified tolerances; sensitivity/specificity at the agreed operating point; and reproducibility within an accepted numerical tolerance under containerized execution).

The bounds of acceptable drift and data integrity requirements (examples): performance must not drop by more than 5% absolute on sentinel tests; control charts must remain in-control; metadata completeness and checksum verification must pass; and any detected domain shift beyond thresholds triggers review and, if needed, partial revalidation.

Adapting PCCPs for Toxicology: We propose that the OECD and US EPA adopt a similar “*Toxicological PCCP*” framework for AI-based NAMs. In this model, the initial validation dossier would include a “*Maintenance and Evolution Plan*.” As long as the Post-Validation AI Companion (see Section 5.5) demonstrates that the updated method remains within the pre-approved performance bounds defined in the PCCP, the method would retain its validated status without requiring a new Council Decision or regulatory review.

This shift requires a move away from “*regulate-and-forget*” toward an “*adapt-and-learn*” regulatory culture. By validating the process of change rather than just the state of the art, regulators can harness the accuracy of evolving AI tools without sacrificing the legal certainty required for chemical safety assessment.

## 8 Future perspectives and opportunities

The future of validation in toxicology presents unprecedented opportunities for transformation through emerging technologies and novel approaches. This evolution will fundamentally change how we evaluate and implement new methods while enhancing our ability to protect human health and the environment. We described aspect of validation of DNT assays in particular earlier (Smirnova et al., 2024; Celardo et al., 2025; Cöllen et al., 2025; Hartung and Smirnova, 2025) and brain organoids for this in particular (Smirnova and Hartung, 2024; Alam El Din et al., 2024). Box 2 describes a Hypothetical Case Study, how NGV could be applied to example of a Developmental Neurotoxicity (DNT) Organoid Assay.

Box 2 **Worked example (illustrative) – NGV of a brain organoid-based DNT assay.**

Context of use (fit-for-purpose): screening and prioritization of potential developmental neurotoxicants, with outputs expressed probabilistically (e.g., probability of DNT hazard at defined exposure ranges). The steps below illustrate how NGV differs from classical validation by making acceptance criteria explicit, quantifying uncertainty, and planning for lifecycle monitoring.

1. Reference set design (decision-ready coverage): Define a balanced positive/negative reference set spanning relevant modes of action (e.g., neurite outgrowth inhibition, synaptogenesis disruption, glial maturation, endocrine-mediated neurodevelopmental perturbation). Acceptance criterion (example): chemical-space and mechanism coverage review confirms no single MoA dominates; negatives include “near-miss” stressors that challenge specificity.
2. Study design simulation (power and variability): Use historical variability (donor-to-donor, batch, plate) to simulate study designs and pre-register the design. Acceptance criterion (example): ≥90% power to detect a pre-defined minimal effect size on primary endpoints at the intended operating point, without inflating false positives.
3. Endpoint definition and analytical pipeline lock: Specify primary endpoints (e.g., morphometric signatures, electrophysiology, transcriptomic modules mapped to AOP key events) and lock preprocessing/analysis code (containerized). Acceptance criterion (example): computational reproducibility demonstrated by bitwise-identical outputs for the locked pipeline.
4. Performance metrics with uncertainty: Evaluate predictive performance on an external holdout (ideally multi-site) using pre-specified metrics. Acceptance criteria (example): sensitivity and specificity each ≥0.80 with 95% confidence intervals; calibration metrics reported and within pre-defined bounds; explicit out-of-domain handling.
5. Mechanistic anchoring (AOP/MOA plausibility): Confirm that the assay perturbs expected key events for reference positives (e.g., transcriptomic pathway activation consistent with known AOPs) and does not systematically flag negatives via unrelated stress pathways. Decision point: if performance is adequate but mechanistic plausibility is weak, the method may be restricted to “phenotypic screening” rather than mechanistic decision support.
6. Documentation and evidence package: Generate an NGV dossier containing SOPs, critical parameter ranges, QC/control charts, reference set rationale, and an external validation report with uncertainty. Decision point: qualification/acceptance depends on whether the dossier supports the intended regulatory consequence.
7. Post-deployment monitoring (lifecycle): Implement control charts and drift detection (e.g., reagent lots, donor mix, imaging hardware). Trigger examples: >5% absolute drop in sentinel-set performance or sustained control drift prompts investigation and (if within a PCCP) a constrained update; otherwise, partial revalidation is initiated.

The NGV framework is not limited to *in vitro* or MPS; it is equally critical for data-driven *in silico* approaches that seek to refine and reduce animal use (Hartung and Kleinstreuer, 2025). A prime example is the validation of Virtual Control Groups (VCGs) (Golden et al., 2024; Steger-Hartmann et al., 2020) currently being spearheaded by the IHI VICT3R consortium^1^ (Steger-Hartmann et al., 2025). Unlike traditional validation, which might assess a static method, the validation of VCGs requires a continuous, data-centric approach that aligns perfectly with the principles of e-validation and AI-driven monitoring.

In the VICT3R context, the “method” being validated is an algorithmic process: either statistical matching of historical controls or the generation of synthetic controls via generative AI. Box 3 provides an illustrative NGV workflow that goes beyond reproducibility checks to evaluate biological plausibility of virtual cohorts, robustness to hidden confounders (e.g., genetic drift and husbandry changes), and lifecycle monitoring. This type of evidence can complement ongoing qualification discussions by demonstrating that replacing Concurrent Control Groups (CCGs) with Virtual Control Groups (VCGs) preserves decision-relevant operating characteristics (e.g., type I error control and power) under defined conditions.

The validation approach of VICT3R is only shaping in close discussion with the medical agencies on both sides of the Atlantic. Adherence to NGV principles may additionally support qualification activities. In contrast to validation, qualification of NAMs aims to demonstrate that a given method is suitable for use within a defined regulatory or decision-making context (Harrill et al., 2024). Within VICT3R, this entails providing evidence that VCGs are suitable for use in the safety assessment of new drug candidates or chemical entities. While qualification does not necessarily require that a method has undergone full formal validation, the application of NGV approaches to the generation and use of VCGs would further support ongoing or future qualification efforts. After providing reasonable validation and qualification results, the VCG concept has the potential to reduce 15%–20% of animal numbers, in some cases of non-human primate studies even up to 30% (Steger-Hartmann and Clark, 2023).

Box 3 **Worked example (illustrative) – NGV of virtual control groups (VCGs) within VICT3R.**

Context of use (fit-for-purpose): replacing concurrent control groups (CCGs) with VCGs for specific study designs (e.g., 28-day repeated-dose toxicity studies in rats) to reduce animal use while preserving decision-relevant statistical properties. The workflow below illustrates how NGV operationalizes validity via operating characteristics, uncertainty, and lifecycle monitoring.

1. Data governance and confounder control: curate the historical-control “data lake” with standardized metadata (laboratory, study design, animal strain/substrain, husbandry, anesthesia, analytical platforms). Acceptance criterion (example): confounder-aware filtering reduces unexplained variance and yields stable baselines across time; excluded clusters are documented and auditable.
2. Retrospective simulation to quantify operating characteristics: re-analyze a large set of legacy studies by replacing the observed CCG with matched VCGs (multiple iterations, Z) to estimate the distribution of treatment-effect estimates and uncertainty. Acceptance criteria (examples): type I error inflation remains within a pre-specified margin; non-inferiority of power for detecting effects of concern; and effect-size estimates remain within agreed equivalence bounds stratified by lab and substrain.
3. Synthetic augmentation (if needed) with biological plausibility checks: when data are sparse, generative models may create synthetic controls. Decision point: synthetic augmentation is acceptable only if multivariate dependencies are preserved (e.g., clinical chemistry correlations with histopathology) and synthetic data do not create implausible “physiologies” under expert review.
4. Documentation and transparency: provide a VCG “algorithm card” describing matching variables, model assumptions, dataset versions, and uncertainty propagation, plus a validation report demonstrating operating characteristics and applicability boundaries. Decision point: qualification depends on whether the evidence supports the specific study type and endpoints for which VCGs are proposed.
5. Lifecycle monitoring and change control (PCCP-aligned): continuously monitor baseline drift (e.g., body weight, hematology, clinical chemistry) and covariate shifts over time. Trigger examples: statistically significant drift beyond thresholds prompts (i) investigation, (ii) constrained update within the PCCP, or (iii) partial revalidation if outside the approved change protocol.

AI will increasingly drive innovation in validation approaches (Luechtefeld and Hartung, 2025). Beyond its current applications, AI will enable more sophisticated analysis of validation data, automated identification of potential issues, and real-time optimization of validation protocols. Machine learning algorithms will continue to improve in their ability to identify patterns and relationships in complex datasets, enhancing our understanding of method performance and applicability. Natural language processing will revolutionize how we extract and synthesize information from scientific literature, enabling more comprehensive evaluation of mechanistic relevance and biological plausibility. The development of explainable AI will address current limitations in model interpretability, making AI-based approaches more acceptable for regulatory applications.

The integration of big data represents another frontier in validation advancement. As the volume and variety of toxicological data continue to grow, new approaches for data integration and analysis become essential. Cloud computing and distributed processing will enable handling of massive datasets while maintaining accessibility and usability. Advanced analytics platforms will provide real-time insights into method performance across different contexts and applications. The development of sophisticated data architecture will support seamless integration of diverse data types, from traditional toxicology endpoints to high-dimensional omics data.

Dynamic validation approaches will replace static, one-time assessments with continuous evaluation and refinement of methods. These approaches will enable real-time monitoring of method performance, automatic identification of potential issues, and rapid implementation of improvements. Validation will become an ongoing process rather than a discrete event, with methods continuously evaluated against new data and updated scientific understanding. This dynamic approach will ensure methods remain current and reliable while reducing the time and resources required for validation.

Continuous learning systems represent a paradigm shift in how validation evolves over time. These systems will automatically incorporate new data and findings, update validation assessments and identify areas for improvement. Machine learning algorithms will continuously refine their understanding of method performance and applicability, enabling more accurate predictions of reliability across different contexts. The integration of feedback loops will allow rapid identification and correction of potential issues, ensuring maintained method performance over time.

Personalized risk assessment enabled by advanced validation approaches will transform how we evaluate chemical safety for different populations and individuals. Validation frameworks will evolve to consider genetic variation, life-stage differences, and other factors affecting individual susceptibility. This personalization will enable more accurate prediction of chemical effects across diverse populations while maintaining practical applicability for regulatory purposes. The integration of biomarker data and other individual-specific information will enhance the relevance and accuracy of safety assessments.

Advanced computational modeling will increasingly support validation processes. These models will enable simulation of complex biological systems, prediction of method performance under different conditions, and optimization of validation protocols. The integration of physiologically based pharmacokinetic (PBPK) modeling with *in vitro* methods will enhance our ability to extrapolate between different test systems and to human outcomes. Quantum computing may eventually enable modeling of complex biological processes at unprecedented scales.

International collaboration will accelerate validation advancement through shared resources and expertise. Cloud-based platforms will enable real-time collaboration across organizations and geographical boundaries. Standardized approaches to data sharing and method evaluation will facilitate mutual recognition of validation results between different jurisdictions. The development of international centers of excellence will provide shared resources and expertise for validation activities.

Regulatory frameworks will evolve to accommodate these advancing capabilities while maintaining necessary standards for public health protection. New approaches to method evaluation will enable more rapid regulatory acceptance while ensuring scientific rigor. The development of clear criteria for evaluating AI-based methods will facilitate their integration into regulatory frameworks. Harmonized international standards will support consistent implementation across different jurisdictions. Gourmelon et al. (2024) offer a policy- and practice-oriented analysis of why the uptake of NAMs into regulatory guidelines remains limited, despite robust development pipelines. They argue that validation is undervalued and underfunded, especially in academic contexts, leading to a persistent disconnect between method innovation and regulatory readiness. Their work emphasizes the need for a shared validation infrastructure, including cost-sharing across stakeholders (industry, regulators, NGOs), better documentation of transferability, and realistic budgeting of human and technical resources. The paper also challenges the conventional over-reliance on ring trials, suggesting that well-documented transfer to one or two external labs may, in many cases, be sufficient for demonstrating reproducibility. Importantly, they call for broader recognition of validation as a shared societal investment, highlighting that the annual savings from OECD’s Mutual Acceptance of Data (MAD) are estimated at over €300 million, a strong argument for proactive validation funding. These insights directly support our vision of next-generation validation as an inclusive, iterative, and resource-aware enterprise.

Education and training will transform through advanced technologies and approaches. Virtual reality and augmented reality will enable immersive training experiences for complex methods. Adaptive learning systems will provide personalized education tailored to individual needs and learning styles. Continuous assessment of competency will ensure maintained quality in method implementation across different laboratories and users.

The integration of sustainability considerations into validation frameworks represents another important opportunity (Bernhard et al., 2023). Green chemistry principles will increasingly influence method development and validation, promoting more environmentally sustainable approaches to toxicity testing (Maertens and Hartung, 2018; Maertens et al., 2024b). The evaluation of method sustainability will become an integral part of validation assessments.

These future perspectives and opportunities suggest a transformation in how we approach validation in toxicology. Success in realizing these opportunities requires careful attention to implementation challenges while maintaining focus on the ultimate goal of enhancing public health protection. Through systematic development and implementation of these advancing capabilities, the field can move toward more efficient, accurate, and sustainable approaches to chemical safety assessment.

## 9 Recommendations and path forward

The successful implementation of next-generation validation requires a coordinated and strategic approach across multiple stakeholders. This section outlines specific recommendations and defines a clear path forward for transforming validation practices in toxicology.

Key steps for implementation begin with establishing foundational infrastructure and frameworks. Organizations must first develop robust data management systems capable of handling diverse data types and supporting advanced analytics. Implementation of standardized data formats and quality control processes provides the foundation for more sophisticated validation approaches. Development of clear governance structures and decision-making frameworks ensures consistent application of new validation principles. These foundational elements should be established through collaborative efforts involving multiple stakeholders to ensure broad applicability and acceptance.

Research priorities must focus on addressing critical gaps in current validation capabilities. Development of improved methods for uncertainty quantification and integration of probabilistic approaches represents an immediate priority. Research into artificial intelligence applications for validation should emphasize both the development of new capabilities and enhancement of model interpretability. Investigation of biomarker-based approaches and mechanistic validation frameworks requires sustained attention. Studies examining the relationship between *in vitro* results and human outcomes will strengthen the biological relevance of validation assessments.

Stakeholder roles and responsibilities must be clearly defined to ensure coordinated progress. Regulatory agencies should lead the development of updated guidance documents and frameworks for evaluating new validation approaches. Industry partners must contribute expertise and resources while helping ensure practical applicability of new approaches. Academic institutions should focus on advancing the scientific understanding underlying validation methodologies. International organizations play crucial roles in harmonizing approaches across different jurisdictions. Method developers must engage early with regulatory authorities and provide transparent documentation of validation processes.

While many technical advances have aimed to improve the validation of NAMs, Mondou et al. (2021) emphasize that validation must also be viewed as a sociopolitical process requiring shared infrastructure, trust, and global coordination. Their expert Delphi study identified a lack of consensus on the definition and scope of validation, highlighting the need for standardized data reporting, regulatory engagement, and training. Crucially, they propose the creation of a global “orchestrator” to harmonize efforts and foster voluntary alignment across jurisdictions. These insights suggest that successful implementation of next-generation validation frameworks, including e-validation and AI-driven methods, will depend not only on technical innovation but also on a concerted effort to build a cohesive regulatory science ecosystem.

Implementation timelines should follow a phased approach, allowing for systematic development and evaluation of new capabilities. The initial phase focuses on establishing basic infrastructure and frameworks, typically requiring 12–18 months. Development and testing of advanced validation capabilities follow, with full implementation of sophisticated systems expected within 3–5 years. Regular assessment of progress and adjustment of timelines ensures maintained momentum while accommodating emerging challenges and opportunities.

Regulatory considerations remain paramount throughout implementation. Early engagement with regulatory authorities helps ensure new approaches meet regulatory requirements while maintaining necessary standards for public health protection. Development of clear criteria for evaluating new validation approaches supports consistent regulatory review. Creation of mechanisms for updating regulatory guidance enables incorporation of advancing capabilities while maintaining regulatory confidence. International harmonization efforts must consider varying regulatory requirements across different jurisdictions.

Technical implementation requires careful attention to system architecture and capabilities. Development of modular platforms allows organizations to adopt capabilities gradually while maintaining system integrity. Implementation of robust security measures protects sensitive data while enabling appropriate sharing. Regular system updates ensure maintained functionality and incorporation of advancing technologies. Creation of user-friendly interfaces supports broad adoption across different stakeholder groups.

Education and training programs must evolve alongside technical capabilities. Development of comprehensive training materials supports consistent implementation across different organizations. Implementation of competency assessment systems ensures maintained quality in method application. Regular updates to training programs reflect advancing capabilities and emerging best practices. Creation of user support systems provides ongoing assistance during implementation.

Resource allocation requires careful consideration of both immediate and long-term needs. Investment in infrastructure development provides necessary foundation for advanced capabilities. Allocation of resources for ongoing maintenance and updates ensures sustained functionality. Development of shared resources and collaborative platforms enhances accessibility while reducing individual organization costs. Creation of funding mechanisms supports continued advancement of validation capabilities.

Success metrics must be clearly defined and regularly assessed. Development of quantitative performance indicators enables objective evaluation of progress. Implementation of monitoring systems tracks adoption and effectiveness of new approaches. Regular assessment of stakeholder satisfaction ensures maintained alignment with user needs. Creation of feedback mechanisms supports continuous improvement of validation systems.

International collaboration remains essential for successful implementation. Development of harmonized approaches supports consistent application across different regions. Creation of mechanisms for sharing validation results reduces duplication of effort. Implementation of mutual recognition agreements facilitates global acceptance of validated methods. Establishment of international working groups coordinates advancement of validation capabilities.

Looking forward, the path to NGV requires sustained commitment from all stakeholders. Regular review and updating of implementation plans ensure maintained relevance and effectiveness. Development of mechanisms for incorporating emerging technologies enables continued advancement of capabilities. Creation of sustainable funding models supports long-term development and maintenance of validation systems. Establishment of centers of excellence provides ongoing support for implementation efforts.

Through careful attention to these recommendations and coordinated effort across stakeholders, the field can successfully transition to next-generation validation approaches. This transformation will enable more efficient and accurate safety assessment while maintaining necessary standards for public health protection.

## 10 Conclusion

The transformation of validation approaches in toxicology represents a critical turning point in our ability to assess chemical safety efficiently and accurately. Throughout this examination of next-generation validation, we have explored how modern technologies, particularly artificial intelligence and advanced computational approaches, can revolutionize the way we evaluate new methods while maintaining scientific rigor and enhancing human health protection.

Next generation validation fundamentally reimagines the validation process, moving beyond simple correlation with animal data to embrace sophisticated understanding of human biology and toxicological mechanisms. The integration of mechanistic validation, biomarker-based approaches, and artificial intelligence creates a more comprehensive framework for method evaluation. This evolution enables validation to keep pace with advancing scientific capabilities while providing the confidence necessary for regulatory acceptance.

The e-validation framework emerges as a promising solution to current validation challenges, offering sophisticated tools for reference chemical selection, study simulation, and ongoing method evaluation. By leveraging artificial intelligence and modern computational capabilities, this approach can significantly reduce the time and resources required for validation while enhancing thoroughness and scientific rigor. The introduction of post-validation companion AI agents represents a particularly innovative advance, enabling continuous monitoring and refinement of validated methods.

Looking to the future, validation will increasingly embrace dynamic, adaptive approaches that evolve alongside scientific understanding and technological capabilities. The integration of artificial intelligence will continue to enhance our ability to analyze complex data, identify patterns, and make informed decisions about method validity. Real-time monitoring and updating of validation status will become standard practice, ensuring methods remain current and reliable over time.

Success in this transformation requires coordinated effort across multiple stakeholders. Regulatory agencies must evolve their frameworks to accommodate new approaches while maintaining necessary standards for public health protection. Industry partners must invest in implementing new capabilities while sharing expertise and resources. Academic institutions must advance the scientific understanding underlying modern validation approaches. International collaboration becomes increasingly crucial for ensuring harmonized implementation across different jurisdictions.

The vision for the future of validation extends beyond simple method evaluation to enable truly next-generation risk assessment. This includes support for integrated testing strategies, probabilistic approaches to uncertainty, and personalized risk assessment considering individual variability. The integration of exposure considerations and real-world relevance will enhance the utility of validated methods for protecting public health.

This transformation carries profound implications for toxicology and public health protection. More efficient and accurate validation enables faster implementation of improved methods for chemical safety assessment. Enhanced understanding of biological mechanisms and human relevance supports better prediction of potential health effects. The reduction in animal testing aligns with both ethical imperatives and scientific advancement.

The path forward demands sustained commitment and investment from all stakeholders. Implementation of necessary infrastructure, development of sophisticated analytical capabilities, and evolution of regulatory frameworks require coordinated effort and resources. However, the potential benefits in terms of enhanced public health protection and more efficient chemical safety assessment justify this investment.

We stand at a crucial moment in the evolution of toxicology, with the opportunity to fundamentally transform how we validate and implement new methods for chemical safety assessment. The tools and approaches outlined in this article provide a roadmap for this transformation, but success requires active engagement and commitment from all stakeholders. The time has come to embrace these advances and work together toward implementation of next-generation validation approaches.

Our call to action extends to all participants in the field of toxicology. Regulatory agencies must take leading roles in developing updated guidance and frameworks for evaluating new validation approaches. Industry partners must invest in implementing new capabilities while sharing expertise and resources. Academic institutions must advance the scientific understanding underlying modern validation approaches. International organizations must coordinate harmonization efforts across different jurisdictions. Only through coordinated effort can we realize the full potential of next-generation validation to enhance chemical safety assessment and protect public health.

The transformation of validation represents not just a scientific and technical challenge, but an opportunity to fundamentally improve how we assess chemical safety and protect human health. By embracing these advances while maintaining necessary scientific rigor, we can create a more efficient, accurate, and ethically sound approach to toxicological method validation. The future of validation begins now, and its success depends on our collective commitment to this transformation.

## Footnotes

1
https://www.vict3r.eu
